# Seroprevalence of high incidence congenital infections among pregnant women in Coatepeque, Guatemala and surrounding areas, 2017–2018

**DOI:** 10.1371/journal.pntd.0011248

**Published:** 2023-04-24

**Authors:** Victoria J. Hicks, César Sánchez, María Reneé López, Anna Gottschlich, Laura M. Grajeda, Amanda Balish, Ana Gómez, Nevis Nuñez, Julio Juárez, Beatriz López, Mariangeli Freitas-Ning, Celia Cordón-Rosales, Manuel Sagastume, John P. McCracken, Andrés Espinosa-Bode, Loren Cadena, Terrence Q. Lo

**Affiliations:** 1 Centro de Estudios en Salud, Universidad del Valle de Guatemala, Guatemala City, Guatemala; 2 Division of Global Health Protection, Center for Global Health, Centers for Disease Control and Prevention, Central America Region Office, Guatemala City, Guatemala; 3 Departamento de Epidemiología, Ministerio de Salud Pública y Asistencia Social, Guatemala City, Guatemala; 4 Global Health Institute, College of Public Health, University of Georgia, Athens, Georgia, United States of America; 5 Division of Global Health Protection, Center for Global Health, Centers for Disease Control and Prevention, Atlanta, Georgia, United States of America; 6 Departamento de Epidemiología, Área de Salud de Quetzaltenango; Ministerio de Salud Pública y Asistencia Social, Quetzaltenango, Guatemala; 7 Hospital Regional “Dr. Juan José Ortega” de Coatepeque, Ministerio de Salud Pública y Asistencia Social, Coatepeque, Guatemala; 8 Unidad de Atención Integral del VIH e Infecciones Crónicas “Dr. Carlos Rodolfo Mejia” del Hospital Roosevelt, Guatemala City, Guatemala; Tulane University School of Public Health and Tropical Medicine, UNITED STATES

## Abstract

Maternal infections during pregnancy can potentially cause birth defects and severe adverse effects in infants. From 2017 to 2018, we investigated the seroprevalence of five antibodies among 436 mother-infant pairs enrolled in a pregnancy cohort study in Coatepeque, Guatemala. Upon enrollment (< 20 weeks gestational age) and shortly after delivery, we measured the prevalence of IgG and IgM antibodies against *Toxoplasma gondii* (*T*. *gondii*), rubella, and cytomegalovirus (CMV) in mothers and newborns and used rapid tests to detect HIV and syphilis (*Treponema pallidum*) in mothers. The mean cohort age was 24.5 years. Maternal *T*. *gondii* IgM and IgG seropositivity was 1.9% and 69.7%, respectively. No women were positive for HIV, syphilis, or rubella IgM. Maternal rubella IgG seropositivity was 80.8% and significantly increased with age. Maternal CMV IgM and IgG seropositivity were 2.3% and 99.5%, respectively. Of the 323 women tested at both timepoints, IgM reactivation occurred in one woman for *T*. *gondii* infection and in eight for CMV. No newborn was seropositive for CMV IgM or rubella IgM. One newborn was seropositive for *T*. *gondii* IgM. Congenital *T*. *gondii* and CMV infections are important public health issues for pregnant women, newborns, and healthcare providers in Coatepeque and Guatemala.

## Author summary

Some infections in pregnant women, known as congenital infections, can result in birth defects and harm in newborns. Adverse outcomes from these infections could include smaller than normal head sizes, other physical abnormalities, low birth weights, preterm births, developmental disabilities, stillbirths, and deaths. During the Zika virus epidemic in 2016, it was discovered that Zika virus is a congenital infection resulting in a global public health emergency. As part of our Zika study in Coatepeque, Guatemala in 2017–2018 we wanted to describe if pregnant women had other congenital infections, namely toxoplasmosis, other pathogens (i.e., syphilis, HIV), rubella, and cytomegalovirus. These are priority pathogens for maternal and child health and the Guatemalan Ministry of Public Health and Social Assistance, and in this region, information is scarce about how common these congenital infections are (with exception of syphilis and HIV that are routinely screened for). Our study aims to provide data that can help inform public health authorities to implement prevention activities and establish other policies for public health practice.

## Introduction

The 2016 Zika virus epidemic and Public Health Emergency of International Concern (PHEIC) generated discussion about Zika virus and other congenital infections that can be transmitted vertically and cause a range of disabilities in fetuses and infants [1–11]. A maternal infection from a group of diseases together known as TORCH (toxoplasmosis, other pathogens, rubella, cytomegalovirus, herpes virus) can cause adverse effects including microcephaly, infant deaths, physical abnormalities, stillborn births, low birth weights, preterm births, and other disabilities for newborns, infants and children [3,6,12–18]. The TORCH “other pathogens” may include HIV, syphilis, and parvovirus [13]. The World Health Organization included TORCH testing in its August 2016 guidelines which recommended serological TORCH testing for newborns and neonates as a part of the clinical assessment of congenital Zika syndrome [19].

The Guatemalan Ministry of Public Health and Social Assistance (MSPAS) recommends that if testing is accessible, pregnant women should be screened at the first prenatal visit for TORCH [20,21]. The full spectrum of TORCH testing may be available to pregnant women in large public hospitals in Guatemala, but many public hospitals do not have testing reagents available. Smaller health facilities in Guatemala typically do not have the full scope of TORCH testing available, but do provide access to HIV and syphilis testing.

Recent studies and systematic reviews have estimated the global prevalence of TORCH [22–28]. Nonetheless, it should be noted that Guatemala and other countries in Central America are often not captured in systematic reviews of TORCH pathogens, as many countries do not have population-level estimates or studies of non-HIV TORCH incidence and prevalence [22,23,28]. Most studies of TORCH pathogens in humans in Guatemala are several decades old; we were unable to find recently published studies investigating TORCH in pregnant women in Guatemala [29–33]. Data on HIV, syphilis, and rubella in Guatemala are more recent [34–36]. Through vaccination, Guatemala has eliminated endemic rubella virus transmission [37,38]. The last confirmed case of rubella occurred in the Quetzaltenango department in 2006, and the last case of congenital rubella syndrome in Guatemala was reported in 2008 in the Escuintla department [39].

We conducted a seroprevalence study to investigate toxoplasmosis, HIV, syphilis, rubella, and cytomegalovirus in pregnant women and newborns participating in a broader cohort study of Zika infection in the departments of Quetzaltenango and San Marcos. Our study’s aim was to provide recent data on antibody seroprevalence. Because we did not investigate herpes infection, we collectively refer to the group of tested infections in this seroprevalence study as “TORC” infections.

## Materials and methods

### Ethical statement

All enrolled patients or their parent/guardian (for those under 18 years of age) gave verbal and written informed consent to participate in the broader pregnancy cohort study and test their samples for the TORC pathogens. Study nurses read standardized statements on study objectives, risks, benefits, and activities with potential participants or parents/guardians for those under 18 years of age. The study nurses verbally explained to women that participation in the study would be voluntary, confidential, and participants could withdraw from parts or all of the study at any time; participants signed a written consent to enroll in the study. Women were offered a small amount of mobile phone credit (approximately $3 USD) for completing a study visit. This study was reviewed and approved by the Guatemala National Health Ethics Committee and the Universidad del Valle de Guatemala Ethics Committee (Protocol No: 158-12-2016) as institutional review boards in accordance with the US Government’s Code of Federal Regulations (45 C.F.R. part 46; 21 C.F.R. part 56).

### Pregnancy cohort study

The women in our seroprevalence study were all enrolled in a prospective cohort study of Zika infection during pregnancy in Coatepeque, Guatemala. Briefly, we enrolled 436 pregnant women, between 16 and 40 years old and of gestational age of less than 20 weeks, from May to November 2017. The women were followed through delivery and their newborns were followed up to three months post-partum or until the end of the study in May 2018. Women were recruited from public antenatal clinics at the *Hospital Regional “Dr*. *Juan José Ortega” de Coatepeque* in Coatepeque, Quetzaltenango, or at public health clinics within a 300 km^2^ study area around this hospital and the Health Center at Las Palmas.

Women with high-risk, ectopic, molar, or multiple pregnancies, as well as women deprived of liberty or residing a distance greater than approximately six kilometers from the two main recruitment sites were excluded. Censoring during the study was due to loss to follow-up, missing data, and study population attrition, including moving to an ineligible area, withdrawal, and spontaneous abortion.

### Specimen collection, laboratory testing, and patient management

Study nurses collected up to five milliliters of blood from enrolled pregnant mothers at baseline and delivery using Vacutainer tubes and a separator gel. Specimens were tested at the laboratory at the *Hospital Regional de Occidente San Juan de Dios* in Quetzaltenango, Guatemala, for Immunoglobulin G (IgG) and Immunoglobulin M (IgM) antibodies against *T*. *gondii*, rubella, and CMV, and for HIV and syphilis infection using rapid tests. Up to three milliliters of blood were collected from newborns at birth using venipuncture and the winged blood collection technique (butterfly); newborn samples were tested for IgM antibodies against *T*. *gondii*, rubella, and CMV.

We transported and processed samples within approximately three days of collection. *Toxoplasma gondii*, rubella, and CMV IgM and IgG antibodies were detected in serum samples using a chemiluminescent microparticle immunoassay on the Abbott Architect i1000SR immunoassay analyzer [40]. The Alere Determine HIV–1/2 Ag/Ab Combo and the Immutrep RPR were used to detect HIV and syphilis antibodies, respectively, in serum samples [41,42]. All procedures were performed according to the manufacturer’s instructions.

Women identified with *T*. *gondii* and CMV IgM positive infections were referred to the *Hospital Regional “Dr*. *Juan José Ortega” de Coatepeque* or the *Centro de Salud de Coatepeque* for additional evaluation and treatment. The *Unidad de Atención Integral del VIH e Infecciones Crónicas “Dr*. *Carlos Rodolfo Mejia” del Hospital Roosevelt*, the national reference facility for infectious diseases located in Guatemala City, performed IgG avidity testing on a select number of *T*. *gondii* and CMV IgM positive baseline samples as per available resources and clinical discretion. Avidity testing results for *T*. *gondii* and CMV IgG were used in conjunction with the IgM serology results to assist in the determination of recency of infections when available.

### Data collection and management

A study nurse collected standardized demographic and risk factor data from enrolled participants using study questionnaires. At baseline, study nurses reviewed any available medical records for participants for documentation indicating a prior TORCH infection. The questionnaire responses, medical chart review, and assessment observations were entered on encrypted electronic tablets using REDCap electronic data capture software [43]. The raw data files were stored securely on servers in Guatemala City.

The outcomes of *T*. *gondii*, HIV, syphilis, rubella, and CMV testing included reactive, nonreactive, indeterminate, or invalid, based on the laboratory testing kits’ defined limits. Samples were excluded from the analysis if there was insufficient volume for testing, missing or invalid laboratory results, or missing linked epidemiological data, unless noted otherwise. In addition to analysis by specimen, we examined the outcome of seroreactivity at either time period.

Home crowding was defined as three or more people per bedroom. Poor housing conditions were defined as having walls, roofs, or floors made of anything other than bricks, metal, or stone. We used two variables to investigate cat ownership: any cat ownership and owning two or more cats compared to just one. Home characteristics and asset ownership were used to calculate socioeconomic status (SES) wealth index quintiles using the same methodology as in the national maternal and child health survey (Encuesta Nacional de Salud Materno Infantil (ENSMI) 2014–2015) [44]. A variable was created to capture formal, salaried employment in mothers.

### Statistical analyses

All data were analyzed using R (v. 3.5.1). To assess differences between variables, we used Chi-square tests of proportions and Fisher’s exact tests to compare categorical variables, t-tests to compare continuous variables, and Cochran–Armitage test for trend to assess linear trends in nominal proportions. To analyze predictors of IgG seroprevalence, crude and adjusted odds ratios with 95% confidence intervals were calculated using logistic regression models. In regression models examining rubella IgG seroprevalence, the five-year age group, education level, previous occupation, and wealth index were analyzed. In regression models examining *T*. *gondii* IgG seroprevalence, the five-year age group, education level, previous occupation, wealth index, poor home condition, primary water source, and cat ownership were analyzed. Included independent variables were socio-demographic variables and risk factors for maternal rubella vaccination and *T*. *gondii* infection based on the published literature.

## Results

### Enrollment and participation

**Fig 1:**
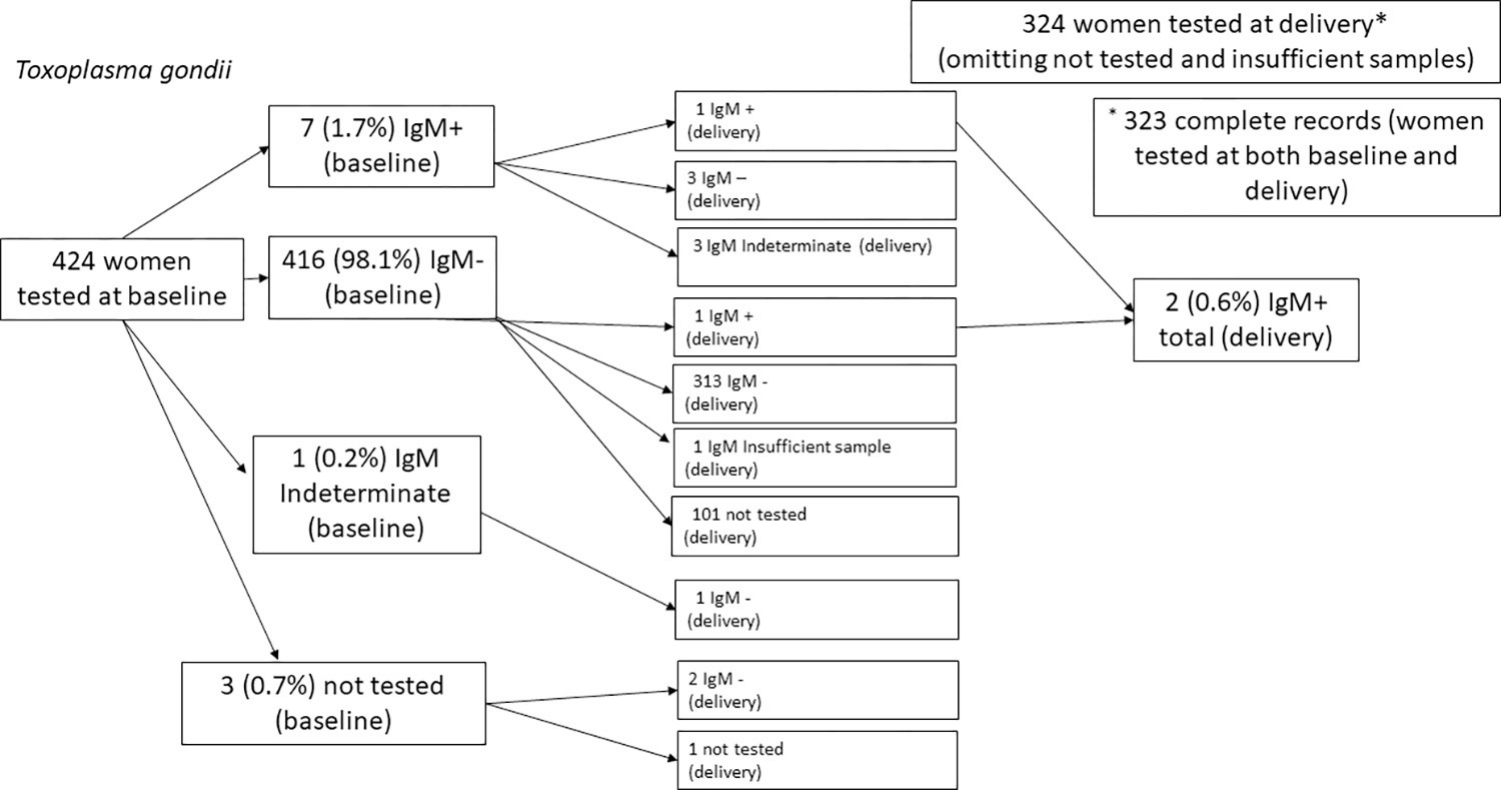
Women were tested at baseline and delivery for IgM antibodies against *Toxoplasma gondii*.

**Fig 2:**
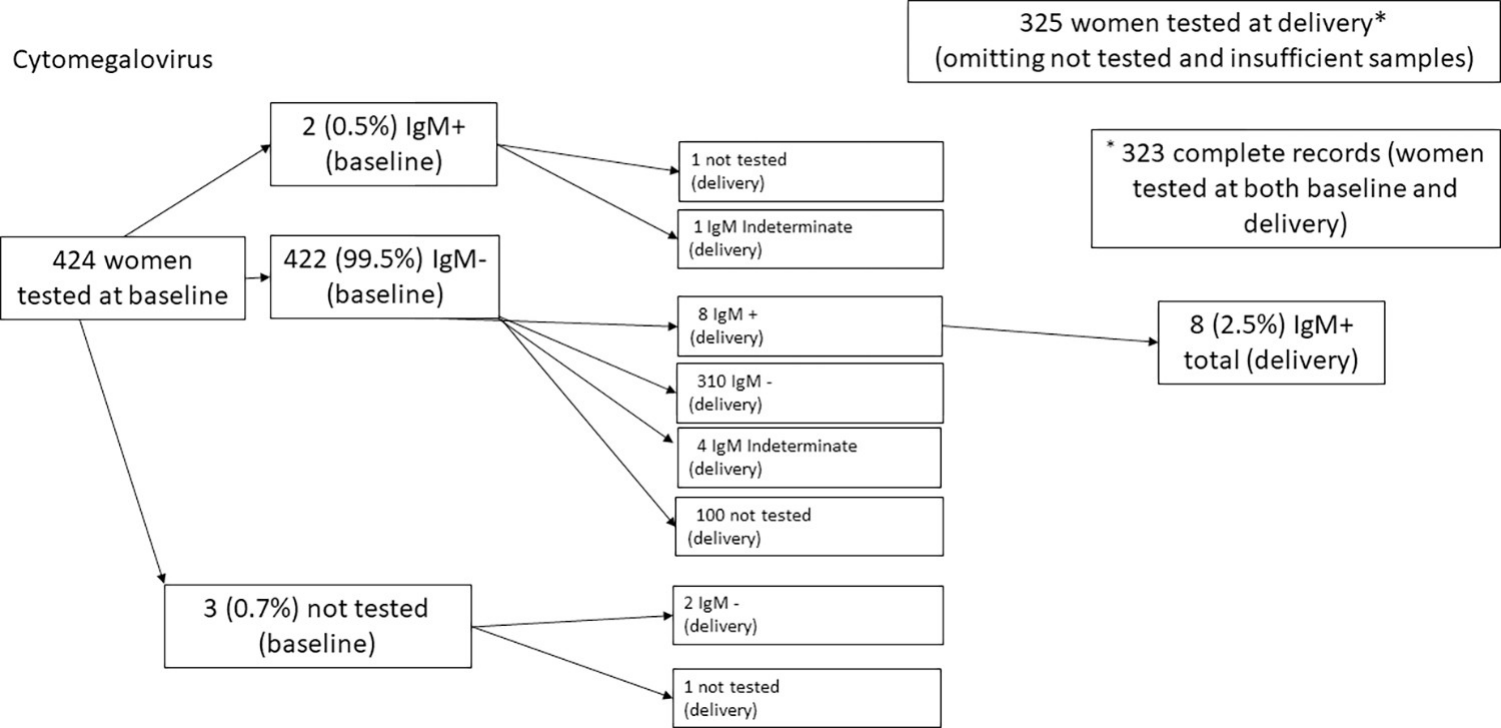
Women were tested at baseline and delivery for IgM antibodies against *Cytomegalovirus*.

At baseline, a total of 424 blood samples from pregnant women were processed for *T*. *gondii*, rubella, and CMV IgG and IgM (Figs 1 and 2). At delivery, we processed 325 participant samples and 231 newborn samples. Overall, 325 women had both baseline and delivery samples. In comparison with the 425 women that were evaluated at baseline only, the women who had samples from both baseline and birth did not significantly differ in age, the highest level of education achieved, wealth index, or baseline positive result from a TORC infection. We were unable to process 60 of the 291 (20.6%) drawn newborn samples due to insufficient sample volume or blood hemolysis. Women with newborns that provided samples did not significantly differ compared to women without newborn samples in age, highest level of education achieved, wealth index, or baseline positive result from a TORC infection.

### Baseline demographic information

Most participants resided in the municipalities of Coatepeque (64.9%) and Pajapita (20.4%), in the departments of Quetzaltenango and San Marcos, respectively. The mean age of the cohort was 24.5 (± 5.3) years (Table 1). Over half (54.2%) of the 413 participants had never attended school or had less than a sixth-grade level education. Primary home drinking water sources were almost evenly divided between bottled water (34.1%), natural water types (32.9%), or municipal water (32.9%). At enrollment, 248 (59.2%) of 419 participants confirmed that they already had at least one child under the age of five. Only four women presented a vaccination card at delivery to the study nurses.

**Table 1:** Baseline Characteristics of Pregnant Women Tested for TORC, Quetzaltenango and San Marcos Departments, Guatemala, 2017–2018 (N = 427).

		N (%)
		**Total (n = 427)**
**Age (years)**	Mean (SD)	24.5 (± 5.3)
	Median (IQR)	24 (20.5–28.0)
**Age group (years)**	16–20	107 (25.1%)
	21–25	151 (35.4%)
	26–30	111 (26.0%)
	31–35	39 (9.1%)
	36–40	19 (4.4%)
**Highest level of education completed** ^**1**^	No formal education	20 (4.8%)
	Some basic education	90 (21.8%)
	Some or completed basic school	99 (24.0%)
	Some or completed primary school	204 (49.4%)
**Wealth quintiles**	Lowest	11 (2.9%)
	Low	83 (21.7%)
	Middle	104 (27.2%)
	High	121 (31.7%)
	Highest	63 (16.5%)
**Quetzaltenango department**		301 (70.5%)
	Coatepeque Municipality	277 (64.9%)
	Flores Costa Cuca Municipality	10 (2.3%)
	Colomba Municipality	14 (3.3%)
**San Marcos department**		126 (29.5%)
	El Quetzal Municipality	14 (3.3%)
	La Reforma Municipality	5 (1.2%)
	Nuevo Progreso Municipality	20 (4.7%)
	Pajapita Municipality	87 (20.4%)
**Primary water source** ^**2**^	Clean	304 (72.6%)
	Unclean	115 (27.4%)
**Previous pregnancies**	Yes	301 (71.0%)
	No	123 (29.0%)
**Number with previous pregnancies resulting in miscarriage (loss before 5 months)**	0	259 (86.0%)
	1	41 (13.6%)
	2	1 (0.3%)
**Number with previous pregnancies resulting in fetal loss (loss after 5 months)**	0	287 (95.3%)
	1	14 (4.7%)
**Number with previous pregnancies resulting in low birth weight infants (less than 5 pounds, 8 ounces)**	0	286 (95.0%)
	1	14 (4.7%)
	5	1 (0.3%)
**Number with previous pregnancies resulting in premature birth infants (during or after 5 months)**	0	294 (97.7%)
	1	7 (2.3%)
**Number with previous pregnancies resulting in live births**	0	21 (7.0%)
	1	127 (42.2%)
	> 1	153 (50.8%)

^1^ Education was categorized based on the highest completed education level with “primary” corresponding up to grade 6 and “basic” to grades 7–9 in the US education system.

2 Clean water was defined as bottled, chlorinated, or boiled water. Primary sources for home drinking water were categorized into bottled water, natural water types (well, river, lake, stream, spring, or rain), and municipal water (piped, trucked, or tanked).

### Seroprevalence results

#### Maternal IgG and IgM seroprevalence

All women were nonreactive for HIV and syphilis antibodies at both baseline and delivery. Of the 424 women with data at baseline, 334 (78.8%) were seropositive for rubella IgG (Table 2). The baseline rubella IgG seropositivity for women aged 16–20 years, 21–25 years, 26–30 years, 31–35 years, and 36–40 years was 60.4%, 82.0%, 87.4%, 84.2%, and 94.7%, respectively. Among 325 women tested at delivery, 247 (76.0%) were seropositive for rubella IgG. Overall, 344 of 426 (80.8%) women tested at baseline or birth were seropositive for rubella IgG (Table 2). None of the 426 women tested at either baseline or delivery were positive for rubella IgM. Among the 323 women with a processed sample at both baseline and delivery, all were seronegative for rubella IgM at both time points, and, thus, none seroconverted for rubella (Table 2).

Among 424 women with baseline blood samples, 289 (68.2%) women were seropositive for *T*. *gondii* IgG. Seven (1.7%) of the baseline samples were seropositive for IgM antibody to *T*. *gondii* (Fig 1 and Table 2). *Toxoplasma gondii* IgG avidity testing was performed on five of the seven baseline IgM-positive samples, and three were determined to be primary infections (Table 3 and Fig 1). At delivery, two (0.6%) of 324 women tested were seropositive for *T*. *gondii* IgM (Table 2 and Fig 1) with one also seropositive for *T*. *gondii* IgM at baseline. Overall, 297 of 426 (69.7%) women tested either at baseline or delivery were seropositive for *T*. *gondii* IgG.

Among 424 women with baseline blood samples 422 (99.5%) were seropositive for CMV IgG (Table 2). Two (0.5%) of 424 baseline samples were IgM positive to CMV (Fig 2 and Table 2); avidity testing was performed, and both were determined to be past infections. At delivery, eight (2.5%) of 325 women were seropositive for CMV IgM; (Table 2 and Fig 2). These eight women had a processed sample at both time points and had a reactivation from seronegative to seropositive for CMV IgM (Table 2 and Fig 2); all eight participants were CMV IgG seropositive at both baseline and birth. Overall, 424 of 426 (99.5%) were seropositive for CMV IgG (Table 2).

One of the 323 (0.3%) women with a processed sample at both time points seroconverted from seronegative to seropositive for *T*. *gondii* IgM (Fig 1); this participant also seroconverted from baseline to delivery for *T*. *gondii* IgG.

**Table 2:** Summary of Toxoplasma, Rubella and CMV IgG and IgM Results in Pregnant Women, Quetzaltenango and San Marcos Departments, Guatemala, 2017–2018 (N = 427).

Laboratory test	Women tested at baseline n reactive/N (%)	Women tested at delivery n reactive /N (%)	Women tested at either baseline or delivery n reactive /N (%)	Women tested at both baseline and delivery n reactive / N (%)
*Toxoplasma gondii* IgM	7/424 (1.7)	2/324 (0.6)	8/426 (1.9)	8/323 (2.5)
*Toxoplasma gondii* IgG	289/424 (68.2)	216/325 (66.5)	297/426 (69.7)	223/323 (69.0)
Rubella IgM	0/424 (0.0)	0/325 (0.0)	0/426 (0.0)	0/323 (0.0)
Rubella IgG	334/424 (78.8)	247/325 (76.0)	344/426 (80.8)	261/323 (80.8)
CMV IgM	2/424 (0.5)	8/325 (2.5)	10/426 (2.3)	9/323 (2.8)
CMV IgG	422/424 (99.5)	324/325 (99.7)	424/426 (99.5)	322/323 (99.7)

**Table 3:** Summary of Toxoplasma, Rubella and CMV IgG and IgM Results in Pregnant Women, Quetzaltenango and San Marcos Departments, Guatemala, 2017–2018 (N = 427).

Laboratory test	IgG seroprevalence at baseline	IgM seroconversions (seronegative to seropositive)	Avidity testing
*Toxoplasma gondii*	289/424 (68.2)	1/323 (0.3)	3/5 (60.0)
Rubella	334/424 (78.8)	0/323 (0)	N/A
CMV	422/424 (99.5)	8/323 (2.5)	0/3 (0)

#### Newborn IgM seropositivity

One out of 227 newborns (0.4%) was seropositive for *T*. *gondii* IgM antibodies at birth. The mother of this newborn had evidence of *T*. *gondii* IgM seroconversion at delivery. No newborns were seropositive for rubella IgM or CMV IgM at birth.

#### Predictors for IgG seropositivity

Adjusted regression models were run only to examine seroprevalence of toxoplasmosis and rubella IgG since nearly all participants were seropositive for CMV and there were no detections of HIV or syphilis.

Maternal age of 21–25 years (adjusted odds ratio (aOR) = 4.33, 95% CI: 1.82, 10.2) and 26–30 years (aOR = 3.94, 95% CI: 1.61, 9.67) versus the referent group of age 16–20 years were significantly associated with maternal rubella IgG seropositivity at baseline. No variables, including cat ownership and clean drinking water sources, were significantly associated with maternal *T*. *gondii* IgG seropositivity at baseline in adjusted models.

## Discussion

This seroprevalence study of congenital infections among a cohort of pregnant women in Coatepeque and surrounding areas in Guatemala, identified maternal *T*. *gondii* and CMV IgM and IgG seropositivity, rubella IgG seropositivity, IgM seroconversion for *T*. *gondii* (1 woman), and IgM reactivation for CMV (8 women). No newborn samples were seropositive for CMV IgM or rubella IgM. One of the newborn samples was *T*. *gondii* IgM seropositive. These results add to the limited literature on TORC diseases in Guatemala and call attention to other infections that can be transmitted from mother to child, with potentially serious negative health effects in these vulnerable populations.

Detection of recent *T*. *gondii* infection during pregnancy is necessary to rapidly initiate treatment and prevent vertical transmission. We detected one case of IgM seroconversion for *T*. *gondii*, with evidence of possible vertical transmission from a mother to her newborn. More than two-thirds of the women in our cohort were seropositive for *T*. *gondii* IgG, indicating a previous infection. *Toxoplasma gondii* IgG seroprevalence was higher than most estimates previously reported in Guatemala, though *T*. *gondii* IgM seroprevalence was similar to that found in a previous study by Urrutia et al. [29,33]. Sinibaldi and de Ramirez reported that *T*. *gondii* seropositivity increased with age in their cohort and women between 24 to 26 years of age were at higher risk of contracting a primary *T*. *gondii* infection compared to the other participants in the study, important considerations given that the mean age of our cohort was 24.5 years. Common sources of transmission for *T*. *gondii* infection include contaminated water, sand, and soil and the consumption of raw or undercooked meat, shellfish, raw milk, and unwashed fruits and vegetables [23,45,46]. But, as we did not collect data on most of these risk factors, we included cat ownership, a weak predictor for toxoplasmosis, in our regression analysis [47].

We detected no women with HIV or syphilis infections, consistent with expectations of low prevalence based on a previously published study of pregnant Guatemalan women [36]. Of note, MSPAS operates a HIV unit in Coatepeque that offers antiretroviral treatment along with public health messaging and some family planning methods to the public. These valuable prevention and epidemiologic surveillance activities for sexually transmitted infections may contribute to maintaining a low prevalence of HIV and syphilis in this population.

Among the investigated TORC infections, only rubella is vaccine-preventable. In the decade prior to the study, MSPAS reported an average rubella immunization coverage of 89.1% (ranging from 64.0% to 104.3%) in the municipality of Coatepeque for the first dose of measles, mumps, and rubella (MMR) vaccine [48]. No rubella cases have been reported in Guatemala in the past decade, and our study found no evidence of IgM seropositivity in women or newborns. Our study noted the youngest age group having lowest rubella immunity. We note that Guatemala had a mass MR vaccination campaign in 2007 targeting 9–39 years old and achieved 99% targeted coverage as part of the rubella elimination initiative [49]; the majority of the youngest age group would not have qualified for this campaign. The threat of importation or re-introduction of rubella and other vaccine preventable diseases (VPDs) remains an important risk, as was evident from an imported measles case in Guatemala in January 2018 and ongoing VPD outbreaks in the region [50–53].

In June 2019, the Pan American Health Organization/World Health Organization (PAHO/WHO) released an epidemiological alert to draw attention to the increasing challenge of sustaining rubella elimination in the Americas region [38]. Our results indicate that nearly a fifth of our cohort of pregnant women lack rubella immunity and thus may be at risk for contracting rubella and vertical transmission to their fetus. Age was a significant predictor of baseline rubella IgG in our cohort. Of concern is the youngest age group of 16-20-year olds, who had the lowest prevalence of rubella IgG. This finding further underscores the critical need to maintain a strong routine immunization program in Guatemala and continue progressing towards at least 95% coverage for the first and second doses of the MMR vaccine [38].

The eight women who were CMV IgM seropositive during the study period were CMV IgG seropositive at both baseline and delivery, which may indicate a reinfection or reactivation of a previous exposure. As Zuhair et al. explain, CMV reinfections and reactivations are commonly seen, due to the often ubiquitous presence of CMV [28]. We observed a seroprevalence for CMV higher than the global seroprevalence and previous seroprevalence estimates for the Americas region [28,54]. We could not find recent comparative seroprevalence data on CMV among pregnant women for Guatemala, but other countries in Latin America have also reported rates exceeding 90% [28,55]. Zuhair et al. reported a wide range for global CMV seroprevalence, from 18–100%, and cited risk factors including low socioeconomic status, cultural factors, and behavioral factors [28], all of which may have played a role in the high CMV seroprevalence observed in our study. Given the potential complications from congenital CMV infection, further investigation is warranted. While our newborn samples were all seronegative for CMV, these results are not conclusive because serological methods are no longer recommended to diagnose congenital CMV infections [56].

We also found that, among seropositive women with test results at both times, more women with *T*. *gondii* IgM tested positive at baseline than at delivery, while the opposite was observed for CMV IgM. CMV infection during pregnancy after baseline testing may be a possible explanation as well as having varying levels of immunosuppression for different pathogens throughout pregnancy [57].

### Study limitations

Our study underscores the complexity of diagnosing and determining the timing of maternal and congenital infections, particularly *T*. *gondii* and CMV [58]. Current 2020 guidance recommends not to test for congenital CMV infections via serological testing; the current standard for diagnosing congenital CMV infections is polymerase chain reaction (PCR) on saliva and confirmatory urine testing [56]. We had incomplete follow-up of mothers at delivery, and thus may have missed some seroconversions and reactivations. Furthermore, we were able to perform avidity testing on only a select number of baseline samples, making incidence calculations infeasible. Multiple types of tests and algorithms have been used to confirm maternal *T*. *gondii* and CMV infections and estimate when primary infections occur. Testing algorithms vary in nature but serological testing for newborns, infants and children born to mothers with suspected or confirmed infection of a TORCH pathogen during pregnancy is no longer recommended [14,15,59–62]. At delivery, almost all participants lacked documentation of prior vaccinations, preventing us from objectively verifying prior vaccination against the rubella virus.

Our cohort study took place in urban and semi-urban municipalities near a major city in southwestern Guatemala, and so our findings are not generalizable to other areas, particularly more rural and indigenous communities. Our findings are applicable to Coatepeque and the surrounding areas, and it should be noted that population-based estimates cannot be inferred from our investigation. We recruited participants exclusively within the public health system and were unable to investigate the pathogens of interest in users of private clinics and the employer-based health system, the Guatemala Institute of Social Security (IGSS). Our primary source of comparative data for the TORC pathogens was the MSPAS Health Management Information System (SIGSA), which has limited data available on the prevalence of these pathogens because not all diseases are nationally notifiable. We acknowledge that there may be other sources of unpublished information on TORCH pathogens in Guatemala.

### Impact and applications of research

Our results support the idea that TORC infections are an important, insufficiently addressed public health issue that have potentially serious consequences for pregnant women and infants [1,63]. With the exception of HIV and syphilis, routine antenatal screening and treatment does not include TORC pathogens in the general public health care system in Guatemala. In response to our study’s findings, the *Hospital Regional “Dr*. *Juan José Ortega” de Coatepeque* established a protocol for TORC testing for high-risk patients when resources were available.

Given our findings, the authors and study investigators suggest four considerations for public health authorities in Guatemala: (1) Standard protocols should be developed for the diagnosis, clinical management, and treatment for *T*. *gondii* and CMV in Guatemala. Moreover, *T*. *gondii* and CMV treatment should be available at the local health services for patients to receive the recommended case management. The MSPAS guidelines and other researchers recommend routine testing of pregnant women for TORCH infections in Zika endemic countries, but without standardized protocols and available treatment options, the adoption of such recommendations by clinicians in Guatemala is likely to be low [16,18]. (2) Establish or strengthen surveillance systems for the TORC pathogens in Guatemala. There are no national *T*. *gondii* or CMV surveillance systems in the public health system, and thus the true burden of these diseases is underestimated and likely undertreated. Additionally, interest has been expressed by MSPAS officials and public health partners for establishing a surveillance system for congenital malformations. Having current and accurate data on these infections and birth defects will assist decision-makers in improving treatment and clinical management for treatment and clinical management (3). As we found no incident HIV, rubella, or syphilis cases in our cohort, we encourage that the country maintains the national prevention activities related to HIV, rubella, and syphilis (4). Lastly, we encourage that prevention activities be implemented or increased to reduce CMV and *T*. *gondii* infections, pursuant to evidence of IgM seroconversion during the study period.
